# Single approach to double-channel core decompression and bone grafting with structural bone support for treating osteonecrosis of the femoral head in different stages

**DOI:** 10.1186/s13018-020-01717-3

**Published:** 2020-05-29

**Authors:** Ju’an Yue, Xiaozhong Guo, Randong Wang, Bing Li, Qiang Sun, Wangyan Liu, Jiao Chen, Yingnan Li

**Affiliations:** Department of Joint Surgery, Aviation General Hospital, Courtyard 3, Anwai Beiyuan, Chaoyang District, Beijing, China

**Keywords:** Single approach, Double-channel, Core decompression, Bone grafting

## Abstract

**Background:**

We created a novel method—single approach to double-channel core decompression and bone grafting with structural bone support (SDBS)—to treat early-stage osteonecrosis of the femoral head (ONFH) by improving the Phemister technique. This study aimed to evaluate the results of SDBS for early-stage ONFH.

**Methods:**

Altogether, 53 patients (73 hips) were treated using SDBS during 2016–2018. Bilateral (20 patients) and unilateral (33 patients = 18 left hips, 15 right hips) ONFH was diagnosed. According to the Association Research Circulation Osseous classification stages, the femoral heads were staged as IIB (*n* = 15), IIC (*n* = 19), IIIA (*n* = 34), IIIB (*n* = 4), and IIIC (*n* = 1). The Harris hip score was used to evaluate the hips’ clinical function, computed tomography to evaluate subchondral fractures, and plain radiography to assess the extent of femoral head collapse.

**Results:**

The average follow-up was 20.71 ± 6.65 months (6–36 months). At the patients’ last follow-up, 4 hips were found to require arthroplasty. Thus, the overall femoral head survival rate was 94.52% (69/73). Also, the overall Harris score (84.44 ± 14.57) was significantly higher than that preoperatively (77.67 ± 14.37) (*P* = 0.000). The combined excellent and good rate (76.71%) was significantly higher than that preoperatively (38.36%) (*P* = 0.000). Imaging showed that 16 femoral heads had some ONFH progression. The average length of stay was 6.15 ± 0.86 days. The average incision measured 2.69 ± 0.30 cm. Intraoperative blood loss was 61.20 ± 4.81 ml. There were no complications during or after the operation.

**Conclusion:**

SDBS is an effective method for treating early-stage ONFH. It is a hip-preserving surgical approach to slow/prevent ONFH progression.

## Introduction

Osteonecrosis of the femoral head (ONFH) is a progressive, destructive disease of the hip joint caused by factors such as hormones, alcohol abuse, and trauma [1]. These factors can directly or indirectly destroy the blood circulation of the femoral head, in which case the survival of bone cells and bone marrow tissue may be affected [2, 3]. If, during this period, there is no effective treatment applied, most patients experience progression to femoral head collapse and hip osteoarthritis, resulting in the need for hip arthroplasty [4].

ONFH often occurs in young patients, and total hip replacement is not an ideal choice for them because they will face the risk of revision in the future. Therefore, an early intervention is very important for them [5, 6]. There are many surgical procedures used to preserve the femoral head, including core decompression, vascularized and non-vascularized bone grafts, various types of osteotomy, and a porous tantalum implant [7, 8]. In 1949, Phemister introduced a new, non-vascularized bone-grafting technique that now carries his name [9]. That procedure is as follows: First, a cone of the bone about 8–10 mm in diameter is removed from the femoral head and neck via a lateral approach to the proximal femur. The necrotic bone in the femoral head is then debrided, and a strut consisting of a cortical graft is inserted to provide subchondral support to the femoral head.

We improved this Phemister technique by using a drill (diameter 10 mm) to decompress the necrotic area via double channels and then remove part of the necrotic bone through the channels. The two channels are then filled with fresh-frozen allograft and demineralized bone matrix, after which a nano-hydroxyapatite/polyamide-66 support rod is inserted into the outer top hole to provide subchondral support. We called this procedure the “single approach to double-channel core decompression and bone grafting with structural bone support (SDBS).” In this study, we reported the short-term efficacy of treating ONFH with this new procedure.

## Materials and methods

The Ethics Committee of our hospital approved this study. All patients agreed to participate in it.

Altogether, 53 patients (8 female, 45 male; mean age 38.43 ± 10.48 years, range 16–58 years; mean body mass index 24.60 ± 3.14 kg/m^2^, range 16.98–33.22 kg/m^2^ at the time of surgery) were treated with SDBS between October 2016 and September 2018 for ONFH. The ONFH was bilateral in 20 patients and unilateral in 33 patients (18 in the left hip, 15 in the right hip). The causes of ONFH included corticosteroids in 22 patients (32 hips), alcohol abuse in 17 patients (23 hips), and trauma in 6 patients (6 hips). It was idiopathic in 8 patients (12 hips). The Association Research Circulation Osseous (ARCO) classification was the basis on which we diagnosed, analyzed, and classified ONFH [1]. The femoral heads were staged as IIB (*n* = 15), IIC (*n* = 19), IIIA (*n* = 34), IIIB (*n* = 4), and IIIC (*n* = 1).

### Primary materials

**Fig. 1:**
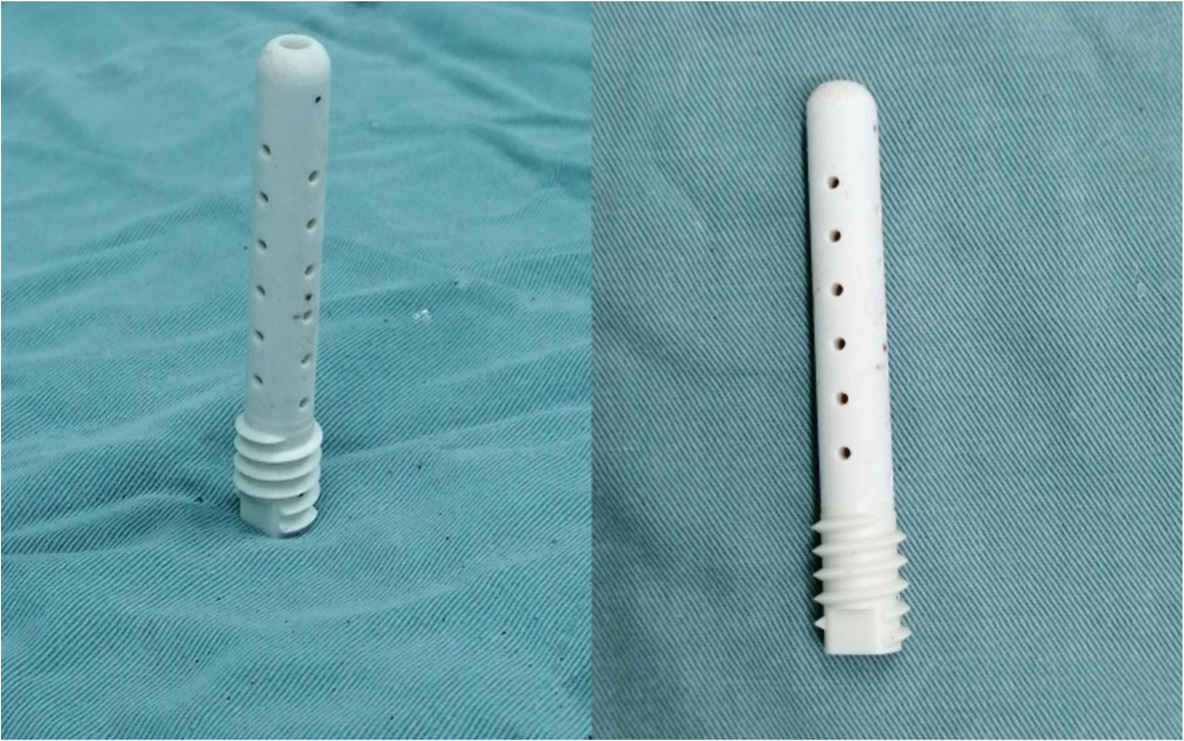
The nano-hydroxyapatite/polyamide-66 (n-HA/PA66) support rod. It is a hollow cylinder (10-mm outer diameter, 4-mm inner diameter) with several holes around the outside

The primary materials included nano-hydroxyapatite/polyamide-66 support rods (Sichuan National Nanotechnology Co., Ltd., Chengdu, China) (Fig. 1), fresh-frozen allograft (FFA) (Shanxi AoRui Biological Material Co., Ltd, Taiyuan, China), and demineralized bone matrix (DBM) (Wright Medical Technology, Inc., Memphis, TN, USA).

### Surgical technique

**Fig. 2:**
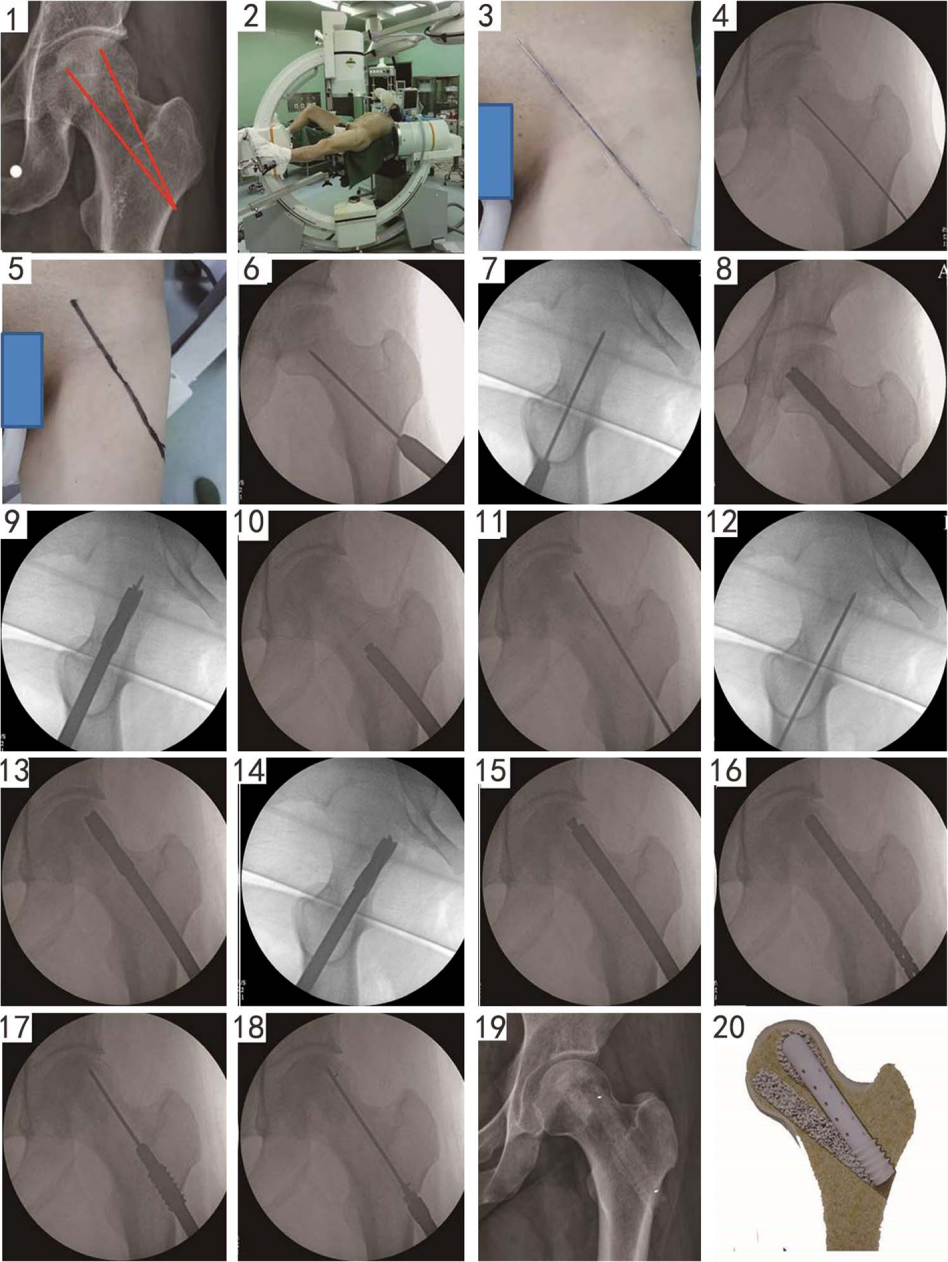
Steps in the surgical procedure that are described in the text

All operations were performed by the same senior doctor in the Orthopedic Department of Aviation General Hospital. First, 10 mg FFA mixed with 1 cc DBM was prepared to fill the double channels. We planned the direction of these double channels on plain radiographs (Fig. 2, panel 1). All patients underwent epidural anesthesia, and after it was deemed satisfactory, the patient was fixed in the traction bed (Fig. 2, panel 2). A Kirschner wire was then placed on the body surface to locate the necrotic area and direction of the wire (Fig. 2, panel 3). Fluoroscopy was used to evaluate the direction of the guide wire (Fig. 2, panel 4), which was then marked on the body surface (Fig. 2, panel 5). Routine surgical areas were disinfected, and sterile towels were applied. After selecting the optimal entrance point, the first guide wire was introduced below and inside the necrotic area (Fig. 2, panels 6 and 7), and a 2-cm skin incision was made at the optimal entrance point. Then, a 10-mm cannulized drill bit was used to extend the diameter along the guide wire up to 3 mm below the cartilage (Fig. 2, panels 8 and 9). FFA particles (7.5 mg) were transplanted into the channel from the necrotic area to the normal area (Fig. 2, panel 10). The second guide wire was then introduced into the outer, top necrotic area from the same entrance point (Fig. 2, panels 11 and 12), and a 10-mm cannulized drill bit was used to extend the diameter along the guide wire up to 3 mm below the cartilage (Fig. 2, panels 13 and 14). FFA particles (2.5 mg) were transplanted into the top of the channel (Fig. 2, panel 15), and a measuring stick was used to determine the length of the second channel (Fig. 2, panel 16). After reaming the proximal femur (Fig. 2, panel 17), a suitable support rod was selected to be inserted into the second channel (Fig. 2, panel 18). The area was then irrigated and the incision sutured. The postoperative radiograph is shown in Fig. 2 (panel 19), as is a photograph of the actual product (panel 20).

### Postoperative treatment

Postoperatively, the patients were given celecoxib to relieve pain and enoxaparine 4000 aXa IU for thrombosis prophylaxis for 7 days after surgery. No other medicine was prescribed. Each patient then participated in a strict rehabilitation and training program. One day after the operation, the patients were advised to start walking with protected weight bearing, using two crutches, and to continue this regimen for the next 6 months. Then, from 7 to 12 months, the patients were advised to begin to discard the use of the crutches and to practice walking with full weight bearing. Thus, by 1 year postoperatively, all patients had completely abandoned the need for crutches.

### Efficacy assessment

All patients underwent follow-up evaluations at 6 and 12 months after surgery during the first year and yearly thereafter. The endpoint of the follow-up was determined by the time that elapsed until the conversion to THA. The Harris hip score (HHS) was used to evaluate the hips’ clinical function. HHS criteria are as follows: excellent (HHS ≥ 90), good (≤ 89 to ≥ 80), fair (≤ 79 to ≥ 70), and poor (< 70). Anteroposterior and lateral plain radiography and computed tomography were performed at each follow-up. Computed tomography was used to evaluate subchondral fractures and radiography to assess the extent of the collapse of the femoral head. Radiographic progression of femoral head collapse was evaluated in consideration of the ARCO classification. The collapse depth of the femoral head was measured using a picture archiving and communication system (PACS, version 11.0) [10]. Two orthopedic specialists were responsible for evaluating all radiographs independently.

Other efficacy assessments included the operation time, intraoperative blood loss, length of stay, and postoperative complications. If the patient underwent total hip arthroplasty (THA) after SDBS—regardless of the cause—it was considered a failed result.

### Statistical analysis

All statistical analyses were completed using SPSS version 22.0 (IBM Corp; Armonk, NY, USA). Data are expressed as means ± standard deviations. Comparisons of the hip joints before and after surgery were performed using a paired *t* test to calculate the HHSs prior to surgery and at the last follow-up. Rate comparisons were performed using the χ^2^ test. The life table method was used to estimate the survival rate*. P* < 0.05 was considered to indicate statistical significance.

## Results

**Fig. 3:**
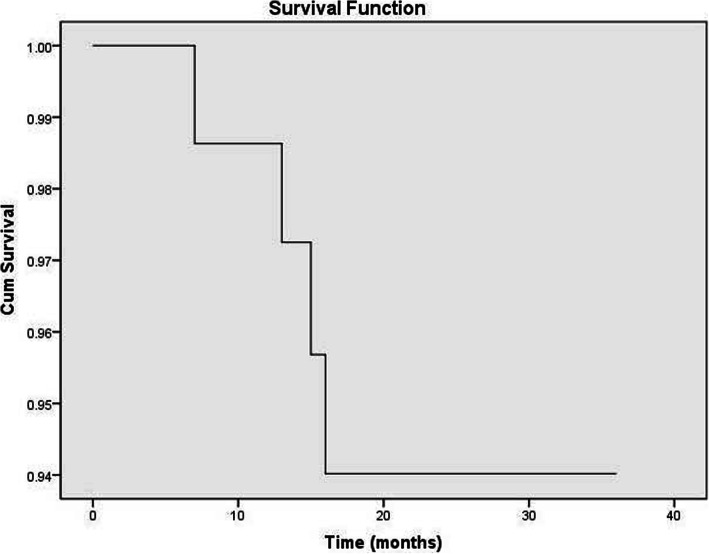
Survival function of SDBS

All patients were enrolled in the study. The average duration of the follow-up was 20.71 ± 6.65 months (range 6–36 months). At the last follow-up, THA was found to be needed for 4 hips, making the survival rate of the femoral head 94.52% (69/72). Details of the four failed hips after undergoing SDBS are shown in Table 1. The estimate of the success rate is shown in Fig. 3.

**Table 1:** Characteristics of clinical failures

*t*	Sex	Age (years)	BMI	Left/right	Risk factors	Pre/last Harris	Pre/last ARCO	Survival time (months)
1	Male	26	23.4	Left	Corticosteroids	65.7/36.4	IIIA/IV	14
2	Male	37	22.0	Right	Corticosteroids	83.7/30.7	IIIA/IV	15
3	Male	30	25.6	Right	Corticosteroids	69/67	IIIA/IIIC	12
4	Male	18	20.0	Right	Trauma	75.7/71.7	IIIC/IV	6

**Fig. 4:**
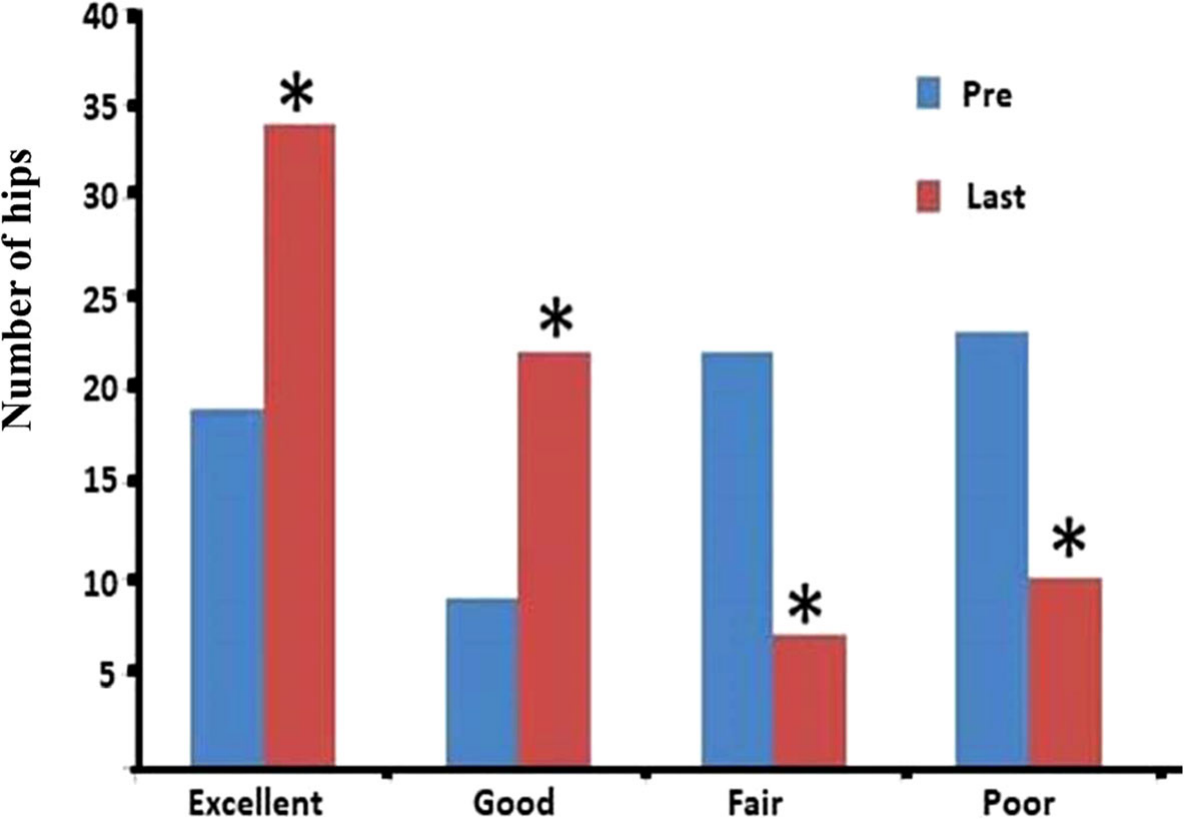
Comparison of the number of hip joints in different stages of clinical efficacy. The asterisk indicates that there is a statistically significant difference (*P* < 0.05) in the composition ratio between the two groups

At the last follow-up, the HHS (84.44 ± 14.57) was significantly higher than that before surgery (77.67 ± 14.37) (*P* = 0.000). Preoperatively, 19 hips were evaluated as excellent, 9 as good, 22 as fair, and 23 as poor. At the last follow-up, the efficacy of SDBS was excellent, good, fair, or poor in 34, 22, 7, and 10 hips, respectively. Preoperatively and at the last follow-up, the number of hip joints at different stages of clinical efficacy is shown in Fig. 4. The combined excellent and good rate at the last follow-up (76.71%) was significantly higher than that before the SDBS operation (38.36%) (*P* = 0.000). The changes in clinical function are shown in Table 2.

**Table 2:** Clinical function of the hips

Pre-function	Last follow-up function			
	Poor (*n*)	Fair (*n*)	Good (*n*)	Excellent (*n*)
Excellent (*n* = 19)	2	1	6	10
Good (*n* = 9)	1	–	3	5
Fair (*n* = 22)	4	4	6	8
Poor (*n* = 23)	3	2	7	11
Total (*n* = 73)	10	7	22	34
Rate (%)	13.70%	9.59%	30.14%	46.58%

**Fig. 5:**
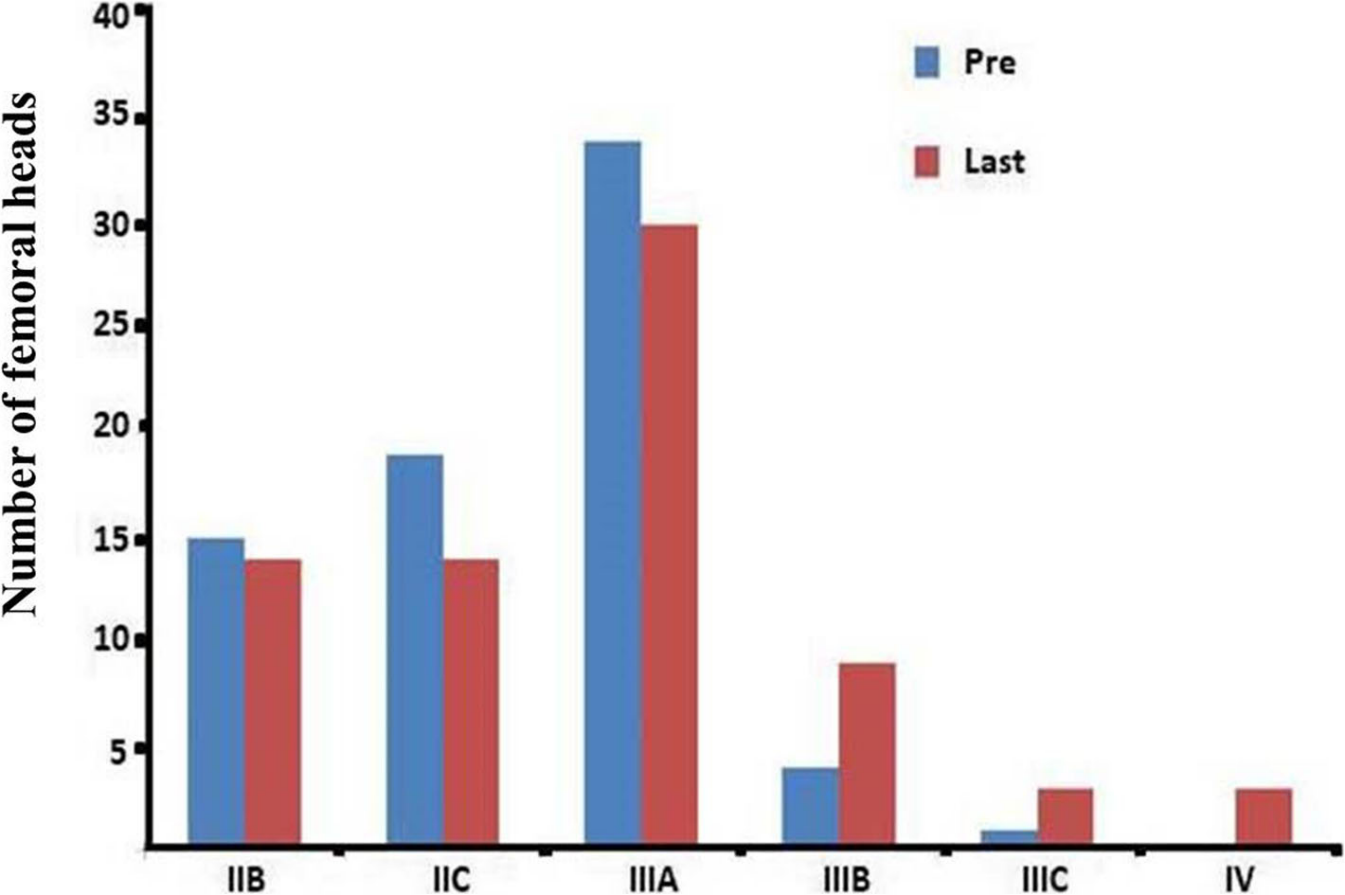
Number of femoral heads at different stages of ARCO

In all, 16 femoral heads showed some progression on their imaging examination. Among the ONFH hips of ARCO stages IIB, IIC, IIIA, IIIB, and IIIC, 1 of 15 hips (6.7%), 5 of 19 hips (26.32%), 7 of 34 hips (20.59%), 2 of 4 hips (50%), and 1of 1 hip (100%), respectively, exhibited progression of femoral head collapse on imaging examinations. Preoperatively and at the last follow-up, the number of femoral heads at different stages of ARCO is shown in Fig. 5. The extents of the progression are shown in Table 3.

**Table 3:** Radiological results

Pre-ARCO		Last follow-up ARCO				
	IIB (*n*)	IIC (*n*)	IIIA (*n*)	IIIB (*n*)	IIIC (*n*)	IV (*n*)
IIB (*n* = 15)	14	-	1	–	–	–
IIC (*n* = 19)	–	14	2	2	1	–
IIIA (*n* = 34)	–	–	27	5		2
IIIB (*n* = 4)	–		–	2	2	–
IIIC (*n* = 1)	–		–	–	–	1
Total (*n* = 73)	14	14	30	9	3	3
Rate (%)	19.18%	19.18%	41.10%	12.33%	4.11%	4.11%

The average length of stay was 6.15 ± 0.86 days (range 5–8 days). The average incision measured 2.69 ± 0.30 cm (range 2.0–3.5 cm). Intraoperative blood loss was 61.20 ± 4.81 ml (range 50–73 ml). No complications (e.g., blood vessel or nerve injury, deep vein thrombosis, wound infection, femoral fracture, rejection) occurred during or after the operation.

**Fig. 6:**
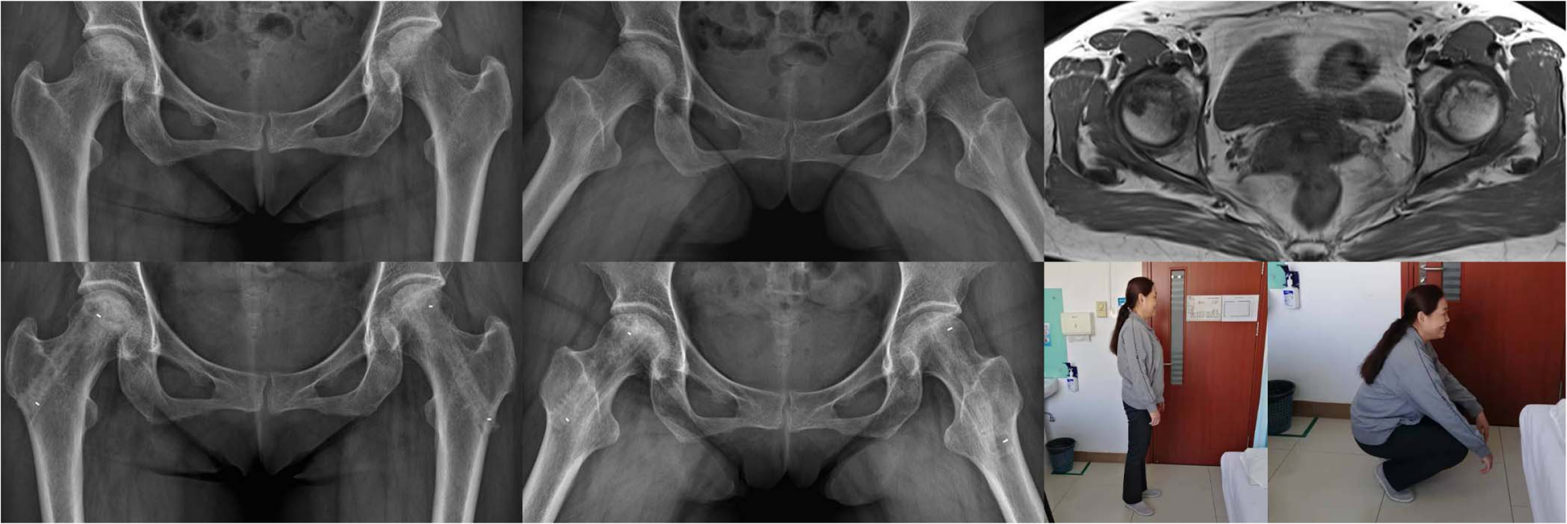
Imaging data before surgery and at the last follow-up. Bottom right, last two panels show the improved patient’s hip function at the last follow-up

A typical case is shown in Fig. 6. The patient was a 55-year-old woman who suffered from steroid-induced ONFH that was diagnosed on preoperative radiographic and magnetic resonance imaging scans (ARCO stage IIB for the left hip and IIIA for the right hip). At the 12-month postoperative follow-up, the necrotic areas of the femoral heads appeared to be repaired, and the function of the hip joint had improved.

**Fig. 7:**
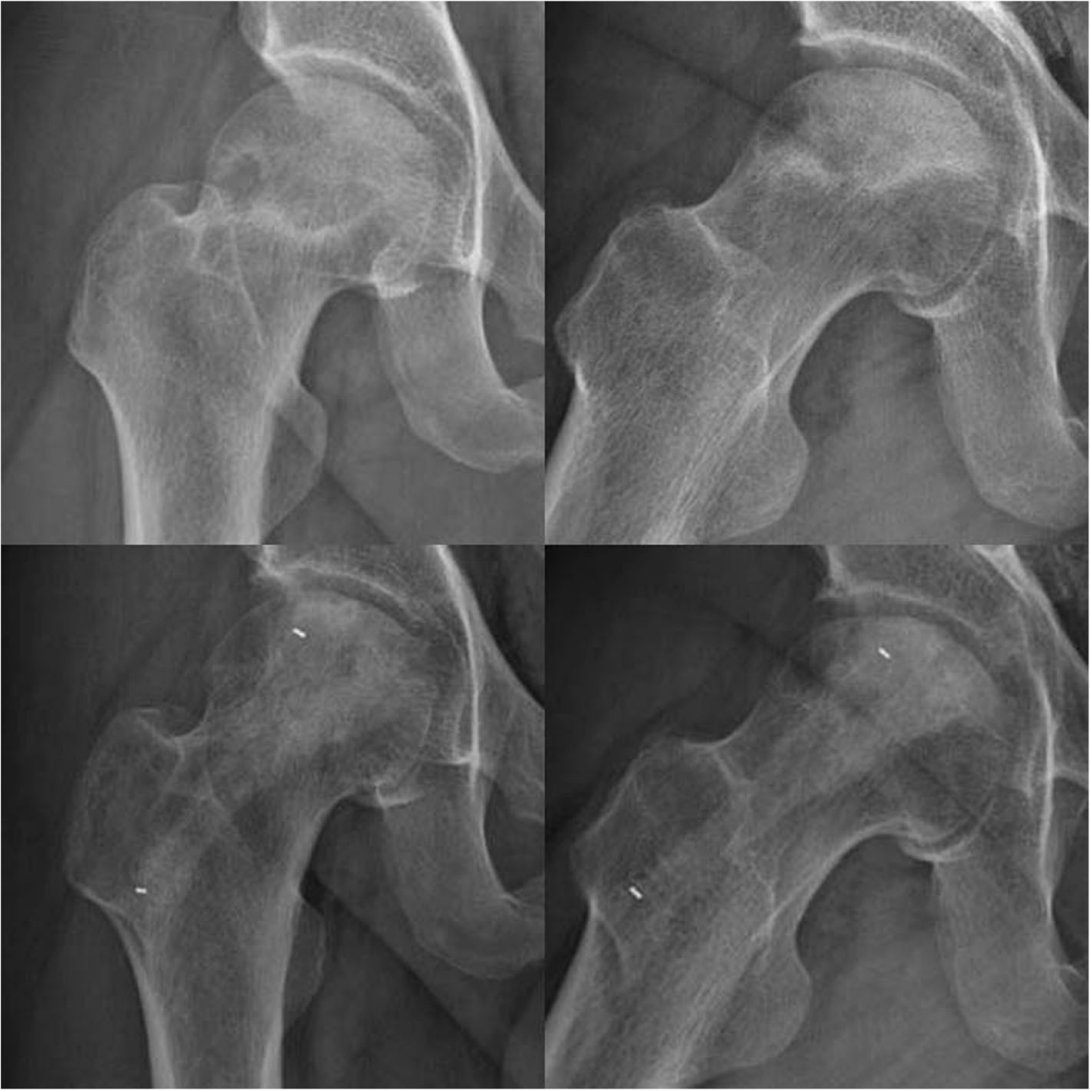
Unilateral radiographs before (top) and at the last follow-up (bottom)

Another typical case is shown in Fig. 7. The patient was a 31-year-old man who suffered from alcohol-induced ONFH. Large areas of osteonecrosis and a meniscus sign are shown on preoperative plain radiographs. At the 8-month postoperative follow-up, the necrotic areas of the femoral heads appeared to be repaired.

## Discussion

After being introduced in 1949, the Phemister technique became very popular during the 1950s and 1960s [11]. A fibular strut graft was first used with this technique, followed by various bone graft materials such as allografts, porous tantalum, vascularized fibula grafts, autogenous bone, and autologous stem cells. Other biomaterials, such as bone morphogenetic proteins, were also widely used in the Phemister technique to treat early-stage ONFH [11–13]. However, Hsu et al. [14] found that 32–42% of their patients failed and required a THA 13–15 months after using the Phemister technique. Keizer et al. found that the 10-year survival was 44% using the same technique [15]. Nonsurgical treatment of ONFH is accomplished using various regimens, including full weight bearing as tolerated, partial weight bearing with crutches, and non-weight bearing. No differences have been reported in the efficacy of these nonsurgical regimens. Nevertheless, nonsurgical treatment for ONFH has been shown in multiple studies to yield extremely poor results, with an overall pooled clinical success rate of 22.7% [16].

The n-HA/PA66 support rod used in this study is composed of nanometer hydroxyapatite (HA) crystal particles evenly dispersed in PA66. HA is an active bioceramic material and the main inorganic component of human and animal bones and teeth. It acts as a scaffold for calcium salt deposition during bone metabolism and can induce new bone formation [17]. PA66 is an organic polymer material with a structure similar to that of collagen. It is easy to process and has strong plasticity [18]. Previous studies have shown that n-HA/PA66 composite biomaterials have good biocompatibility and biosafety, and they can be used to repair bone defects and promote bone growth [19, 20]. Yang et al. [21] used n-HA/PA66 to treat early-stage ONFH and achieved a good effect as it could significantly reduce pain and delay the collapse of the femoral head.

The freeze-dried allograft bone has strong bone conduction ability and is an ideal material or cell carrier for repairing bone defects [22]. In addition, it retains some bioactive bone induction components, so it also has certain bone induction ability [23, 24]. DBM offers a series of chemical methods to remove calcium and fat from the allogeneic bone, reduce immunogenicity, retain bone morphogenetic protein and other osteogenic factors, and play a role in inducing osteogenesis through these osteogenic factors.

Many studies have confirmed that an advanced ARCO stage predicted unsuccessful clinical results [25–27]. We found that, after a thorough follow-up, 7 of 34 hips (20.59%) at ARCO stage IIIA were classified radiographically as exhibiting progression. Although the number of ARCO stage IIIB and IIIC hips is small, the imaging-determined progress was indeed found in 50% (2/4) and 100% (1/1) of cases, respectively. Therefore, we do not recommend SDBS for patients in stage IIIB or IIIC. After reviewing a decade of research on ONFH, Mont et al. [28] also concluded that the last time at which femoral head-preserving surgery can be used successfully in patients with ONFH is at the point of early collapse. We also found early during the follow-up that the clinical effect of SDBS was satisfactory for treating early-stage ONFH.

Many surgeons have used the Phemister technique to treat ONFH with bone grafting and structural support [25]. After a long-term observation, Guo et al. [29] found that there was a contradictory relation between the amount of bone graft and the strength of the support in a single channel. If the bone grafting and structural support were carried out for the necrotic area in the same decompressed channel, a large amount of bone grafting might affect the support strength of the material propping up the femoral head, and insufficient bone grafting might induce osteogenesis. We therefore chose to improve the Phemister technique by implanting a support rod in the outer top channel to provide strong support for the subchondral bone in the weight-bearing area that had become necrotic and to prevent the collapse of the femoral head. We also performed sufficient bone grafting in the inner channels to promote or induce bone formation. The synergy between the support of the outer upper channel and sufficient bone grafting of the inner channel makes up for the contradiction that bone grafting and support weaken one another. Zhou et al. [30] conducted a biomechanical study and found that partially debriding the necrotic area appears to be a better choice for avoiding the collapse of the femoral head. Hence, we did not completely remove the dead bone. The only disadvantage of our technique may be that there is greater radioactive exposure with it than with the Phemister technique.

There are several limitations. First, the sample size was small and the follow-up time short. Large sample and long-term follow-up results are thus needed. Second, risk factors that may lead to surgical failure deserve further analysis.

## Conclusion

SDBS is an effective method for treating early-stage ONFH. It has the advantages of being minimally invasive and offers rapid postoperative recovery, no donor complications, and good recovery of hip function. It represents a new method for treating early-stage ONFH.

## Data Availability

All data and materials used to support the findings of this study are included within the article.

## Abbreviations

ONFH: Osteonecrosis of the femoral head
SDBS: Single approach to double-channel core decompression and bone grafting with structural bone support
ARCO: Association Research Circulation Osseous
FFA: Fresh-frozen allograft
DBM: Demineralized bone matrix
HHS: Harris hip score
PACS: Picture archiving and communication system
THA: Total hip arthroplasty
HA: Hydroxyapatite
